# Clinical isolate characteristics and demographics of patients with *C.jejuni* and *C.coli* infections in Northern Israel, 2015–2021

**DOI:** 10.1017/S0950268823002005

**Published:** 2024-02-05

**Authors:** Ofri Tsafrir, Hanan Rohana, Lior Bousani, Khatib Orsan, Said Abozaid, Maya Azrad, Avi Peretz

**Affiliations:** 1 Azrieli Faculty of Medicine, Bar Ilan University, Safed, Israel; 2 Clinical Microbiology Laboratory, Tzafon Medical Center, Poriya, Israel; 3 The Department of Pediatrics, Tzafon Medical Center, Poriya, Israel

**Keywords:** antimicrobial resistance, campylobacteriosis, *C.coli, C.jejuni*gastroenteritis

## Abstract

*C.coli* is a significant cause of foodborne gastroenteritis worldwide, with the majority of cases attributed to *C.jejuni.* Although most clinical laboratories do not typically conduct antimicrobial susceptibility testing for *C.coli*, the rise in resistant strains has underscored the necessity for such testing and epidemiological surveillance. The current study presents clinical isolate characteristics and demographics of 221 patients with *C.coli* (*coli* and *jejuni*) infections in Northern Israel, between 2015 and 2021. Clinical and demographic data were collected from patient medical records. Susceptibility to erythromycin, tetracycline, ciprofloxacin, and gentamicin was assessed using the standard E-test. No significant correlations were found between bacterial species and patient ethnicity, patient gender, or duration of hospitalization. In contrast, significant differences were found between infecting species and patient age and age subgroup (*P* < 0.001). Furthermore, erythromycin resistance was observed in only 0.5% of the study population, while resistance to ciprofloxacin, tetracycline, and gentamicin was observed in 95%, 93%, and 2.3% of the population, respectively. The presented study underscores the need for routine surveillance of *C.coli* antibiotic resistance.

## Introduction

*C.coli* species are morphologically diverse Gram-negative non-spore-forming bacteria, which appear under the microscope as rod-, curve-, or spiral-shaped [1]. They have been linked to several gastrointestinal conditions, including Barrett’s oesophagus, inflammatory bowel disease (IBD), and bowel cancer [2]. *C. jejuni* is responsible for 80–90% of campylobacteriosis cases, while 10–20% of the cases are caused by *C. coli.* *C. jejuni* has been described as one of the main bacterial pathogens involved in the aetiology of gastroenteritis [3]. Moreover, it has been associated with meningitis, acute cholecystitis, myocarditis, and acute febrile illnesses [1]. While infection can affect individuals of all ages, studies have shown that it is more common in young children, especially those under 2 years of age [4–6]. Transmission occurs via ingestion of contaminated water and food or exposure to infected animals [7]. Despite significant efforts to control infections, *C.coli* has emerged as the primary cause of foodborne illnesses in developed nations [8]. Patients with *C. coli* or *C. jejuni* infections exhibit a variety of symptoms, including watery diarrhoea, fever, and abdominal cramps [2]. While campylobacteriosis is considered a self-limiting disease and does not require specific treatment, antimicrobial treatment is necessary in immunocompromised patients, patients with prolonged symptomology, and patients with coexisting medical conditions. In these cases, macrolides and fluoroquinolones are the most commonly prescribed drugs [9]; however, antibiotic resistance of *C.coli* to these antibiotics is rising [10] and may become insurmountable.

In Israel, *C.coli* morbidity is relatively high, and microbiology laboratories across the country are required to report and submit human isolates to the National C.coli Reference Laboratory, Israeli Ministry of Health, Jerusalem, for further confirmation.

Risk factors for *C.coli* infection in Israel include drinking untreated water, consumption of undercooked food, especially chicken, and exposure to infected animals or their faeces. The Israeli government implemented various strategies to reduce the risk of *C.coli* infection, such as water quality monitoring, promotion of food safety education, and implementation of food safety regulations. However, a 12-year study conducted between 1999 and 2010 revealed a 2.93-fold increase in the annual incidence of all laboratory-confirmed *C.coli* spp. infections in Israel, rising from 31.04 to 90.99 cases per 100,000 population [11].

This work presents a comprehensive analysis of the epidemiological, clinical, and demographic features of *C. jejuni* and *C. coli* isolates collected between the years 2015 and 2021 in Northern Israel. The primary objective was to emphasize the necessity of developing and facilitating effective strategies to prevent and minimize the morbidity caused by these bacteria.

## Methods

### Study design

This study retrospectively analysed records of 221 patients of all ages admitted to the Tzafon Medical Center, Poriya, Israel, between 2015 and 2021 and diagnosed with campylobacteriosis. Clinical and demographic data were collected from the medical records, including age, ethnicity, gender, duration of hospitalization, and symptoms. The type of residence was divided into three categories; in Israel, an area with more than 20,000 inhabitants is usually defined as a city; an area with less than 20,000 inhabitants is defined as a village; and ‘other’ refers to an area of residence that is neither a city nor a village.

The authors assert that all procedures contributing to this work comply with the ethical standards of the relevant national and institutional committees on human experimentation and with the Helsinki Declaration of 1975, as revised in 2008. The study was approved by the Institutional Review Board (IRB) of the Tzafon Medical Center (approval no. POR-0018-22).

### Sample collection and identification

Faecal samples were collected and sent to the clinical microbiology laboratory at the medical centre. Each sample was plated on C.coli-selective agar (BD Diagnostics, Sparks, MD) and incubated at 42 °C for 48 h with a CampyGen™ (Thermo Fischer Scientific, MA, USA) sachet, to ensure microaerobic conditions (85% N_2_, 5% O_2_, 10% CO_2_). *C.coli* colonies were further identified by matrix-assisted laser desorption ionization–time of flight (MALDI–TOF) analysis performed using a Bruker Biotyper system (Bruker Daltonics, Bremen, Germany) ([12]).

### Antibiotics susceptibility testing (AST)

AST for erythromycin, ciprofloxacin, tetracycline, and gentamicin was performed using the E-test method, which determines the minimum inhibitory concentration (MIC) of a specific antibiotic necessary to hinder the growth of bacteria under defined experimental conditions.

After 48 h, several colonies were suspended in brain heart infusion (BHI) broth to create a turbidity of 0.5 McFarland. The suspension was seeded on Brucella blood agar plates (HyLaboratories Ltd., Rehovot, Israel), and then one E-test strip (bioMérieux, Durham, NC) was put on each agar plate for each antibiotic.

The plates were then incubated at 37 °C for 48 h under microaerobic conditions.

*C. jejuni* ATCC 33560 was used as quality control. MIC values were then determined according to the guidelines of the European Committee on Antimicrobial Susceptibility Testing (EUCAST); resistance to tetracycline was defined at MIC >2 mg/L, resistance to ciprofloxacin was defined at MIC >0.5 mg/L, resistance to erythromycin was defined at MIC >4 mg/L for *C. jejuni* and > 8 mg/L for *C. coli*, and resistance to gentamicin was defined at MIC >4 mg/L.

### Statistical analysis

Descriptive statistics of categorical variables are presented as sample size and frequency. The descriptive statistics of continuous variables are presented as mean, median, standard error, and range of values. For continuous variables, the differences between two independent groups were tested using the T test. For categorical variables, the differences between two groups were tested using the chi-squared test or Fisher’s exact test. Statistical significance was defined as a two-sided *p-value* < 0.05. Data were analysed using the RStudio® version 2021.09.0 Build 351.

## Results

### General demographic and clinical data of the study participants, according to bacterial type

This study included records of 221 patients, 82 (37.1%) of whom were females and 139 (62.9%) were males. The patients were Arab (121 (54.8%)) or Jewish (100 (45.2%)), with the majority living in cities (136 (61.5%)), followed by villages (74 (33.5%)). The average age of the study population was 14.82 ± 24.14 years. Thirty samples (13.6%) were infected with *C. coli* and 191 (86.4%) with *C. jejuni.* No differences in demographic characteristics were found between *C. jejuni*-infected patients and *C. coli*-infected patients (Table 1).

**Table 1.** Demographic characteristics of the study population, by bacterial type

Characteristic	*C.coli* (*n* = 30)	*C.jejuni* (*n* = 191)	Total (*n* = 221)	*P-*value
Area of residence, *n* (%)				0.899
City	18 (60.0)	118 (61.8)	136 (61.5)	
Village	10 (33.3)	64 (33.5)	74 (33.5)	
Other	2 (6.7)	9 (4.7)	11 (5.0)	
Gender, *n* (%)				0.724
Female	12 (40.0)	70 (36.6)	82 (37.1)	
Male	18 (60.0)	121 (63.4)	139 (62.9)	
Age, years				0.072
Mean	22.20	13.66	14.82	
SD	29.09	23.14	24.14	
Age subgroups, *n* (%)				0.23
0–2 year	13 (43.3)	91 (47.6)	104 (47.1)	
2–5 years	3 (10.0)	28 (14.7)	31 (14)	
5–18 years	2 (6.7)	29 (15.2)	31 (14)	
18–64 years	6 (20.0)	26 (13.6)	32 (14.5)	
64+ years	6 (20.0)	17 (8.9)	23 (10.4)	
Duration of hospitalization, days				0.068
Mean (SD)	3.50 (1.59)	2.98 (1.43)	3.05 (1.46)	
Ethnicity, *n* (%)				0.574
Arabs	15 (50.0)	106 (55.5)	121 (54.8)	
Jews	15 (50.0)	85 (44.5)	100 (45.2)	

### General demographic and clinical data of the study participants, according to ethnicity

The average age of the Arab patients was 8.06 ± 19.16 years compared to 23.0 ± 26.96 years of the Jewish patients. The group of Arab patients included a significantly higher proportion (65.3%) of children under 1 year of age, while only 25.0% of the Jewish patients belonged to this age group (*p* < 0.001). Significant differences in area of residence (*p* < 0.001) were noted between the Arab and Jewish patients. Out of the total Arab patients, 71 (58.7%) resided in cities, while 65 (65%) of the Jewish patients lived in cities. Furthermore, 50 (41.3%) of the Arab patients lived in villages compared to only 24 (24%) of the Jewish patients who lived in villages. None of the Arab patients lived in areas other than cities or villages, while 11 (11%) of the Jewish patients resided in such areas (Table 2).

**Table 2.** Demographic characteristics of the study population, by patient ethnicity

Characteristic	Arabs (*n* = 121)	Jews (*n* = 100)	Total (*n* = 221)	*P*-value
Area of residence, *n* (%)				**<0.001**
City	71 (58.7)	65 (65.0)	136 (61.5)	
Village	50 (41.3)	24 (24.0)	74 (33.5)	
Other	0 (0.0)	11 (11.0)	11 (5.0)	
Gender, *n* (%)				0.596
Female	43 (35.5)	39 (39.0)	82 (37.1)	
Male	78 (64.5)	61 (61.0)	139 (62.9)	
Age, years				**<0.001**
Mean	8.06	23.00	14.82	
SD	19.16	26.96	24.14	
Age subgroups, *n* (%)				**<0.001**
0–1 year	79 (65.3)	25 (25.0)	104 (47.1)	
1–5 years	21 (17.3)	12 (12.0)	31 (14)	
5–18 years	7 (5.8)	24 (24.0)	31 (14)	
18–65 years	10 (8.3)	22 (22.0)	32 (14.5)	
65+ years	6 (5.0)	17 (17.0)	23 (10.4)	
Duration of hospitalization, days				0.051
Mean	2.88	3.26	3.05	
SD	1.08	1.80	1.46	
Bacterial type, *n* (%)				0.574
*C.coli*	15 (12.4)	15 (15.0)	30 (13.6)	
*C.jejuni*	106 (87.6)	85 (85.0)	191 (86.4)	

Bold= statistically significant

### Resistance rates of C.coli isolates to erythromycin, ciprofloxacin, tetracycline, and gentamicin

**Figure 1.**
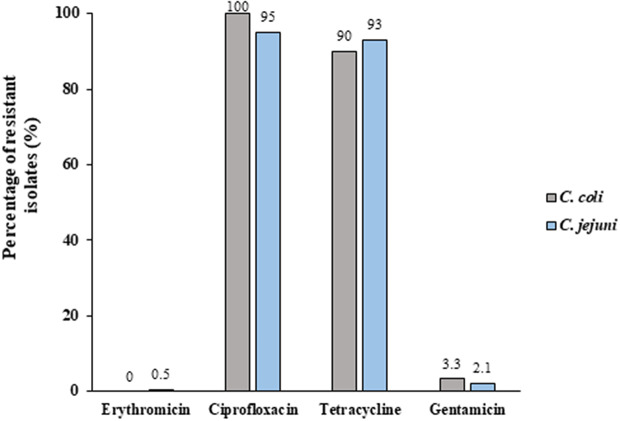
Rates of *C.coli* isolates resistant to erythromycin, ciprofloxacin, tetracycline, and gentamicin. Twenty-one and Two-hundred clinical isolates were tested for antibiotic susceptibility to several antibiotics using the E-test method, which determines the minimum inhibitory concentration (MIC). Resistance was determined according to the EUCAST guidelines.

No significant differences in antibiotic resistance patterns of the two bacteria were observed (Figure 1). Only one *C. jejuni* isolate was resistant to erythromycin (1/221, 0.5%); none of the *C. coli* isolates showed such antibiotic resistance. Five isolates (2.3%) were resistant to gentamicin, one of which (3.3% (1/30)) belonged to the *C. coli* group and 4 of which (2.1% (4/191)) belonged to the *C. jejuni* group. More than 92% of the isolates were resistant to both ciprofloxacin and tetracycline. No significant differences in antibiotic resistance were found between isolates from children (aged 0–18) and from adults (aged ≥18) (data not shown).

### Antibiotic resistance properties of C.coli isolates, categorized by patient ethnicity

Our findings did not reveal any significant dissimilarities in antibiotic resistance between isolates from the Arab and the Jewish patients. The highest antibiotic resistance rate was observed for ciprofloxacin, with 96.7% (117/121) of isolates from the Arab patients and 94% (94/100) of isolates from the Jewish patients showing such antibiotic resistance. The lowest antibiotic resistance rate was observed for erythromycin, with only 0.8% (1/121) of isolates from the Arab patients being resistant and none of the isolates from the Jewish patients (0.0%) showing antibiotic resistance (Table 3).

**Table 3.** Resistance rates of *C.coli*, by patient ethnicity

Antibiotic agent	Arabs (*n* = 121)	Jews (*n* = 100)	Total (*n* = 221)	*P-*value
Erythromycin, *n* (%)				0.36
R	1 (0.8)	1 (1)	2 (0.9)	
S	120 (99.2)	99 (99)	219 (99.1)	
Ciprofloxacin, *n* (%)				0.337
R	117 (96.7)	94 (94.0)	211 (95.5)	
S	4 (3.3)	6 (6.0)	10 (4.5)	
Tetracycline, *n* (%)				0.692
R	113 (93.4)	92 (92.0)	205 (92.8)	
S	8 (6.6)	8 (8.0)	16 (7.2)	
Gentamicin, *n* (%)				0.251
R	4 (3.3)	1 (1.0)	5 (2.3)	
S	117 (96.7)	99 (99.0)	216 (97.7)	

Abbreviations: R, resistant; S, sensitive.

For *C. coli* isolates, the average MICs for erythromycin, ciprofloxacin, tetracycline, and gentamicin were 1.02 μg/mL, 25.07 μg/mL, 175.07 μg/mL, and 1.6 μg/mL, respectively. As for *C. jejuni*, the average MICs for erythromycin, ciprofloxacin, tetracycline, and gentamicin were 3.3 μg/mL, 23.2 μg/mL, 119 μg/mL, and 0.7 μg/mL, respectively.

**Figure 2.**
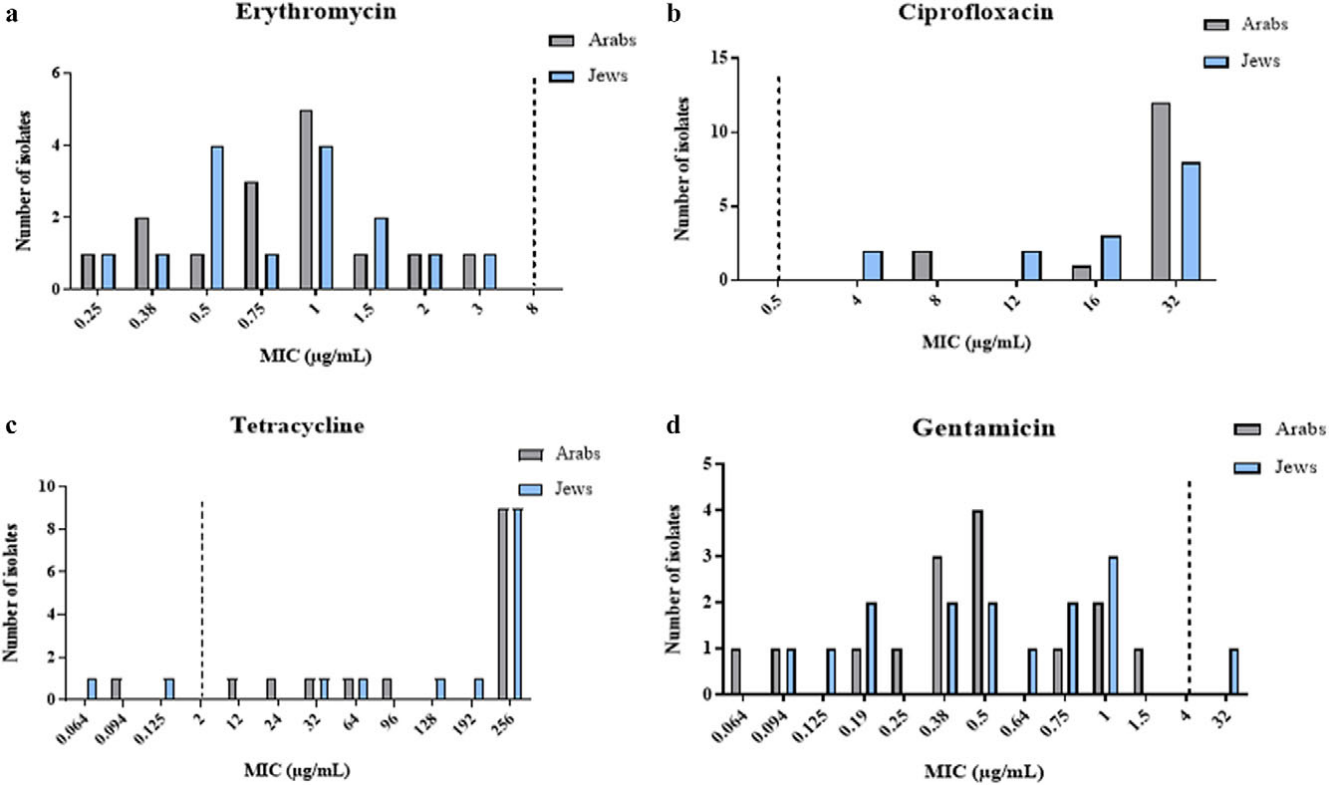
Distribution of minimum inhibitory concentrations (MICs) of antibiotics in *C.coli* isolates, by patient ethnicity. (a) Erythromycin, (b) ciprofloxacin, (c) tetracycline, and (d) gentamicin. The figure presents the antibiotic susceptibility of 30 *C. coli* isolates based on the E-test method.

**Figure 3.**
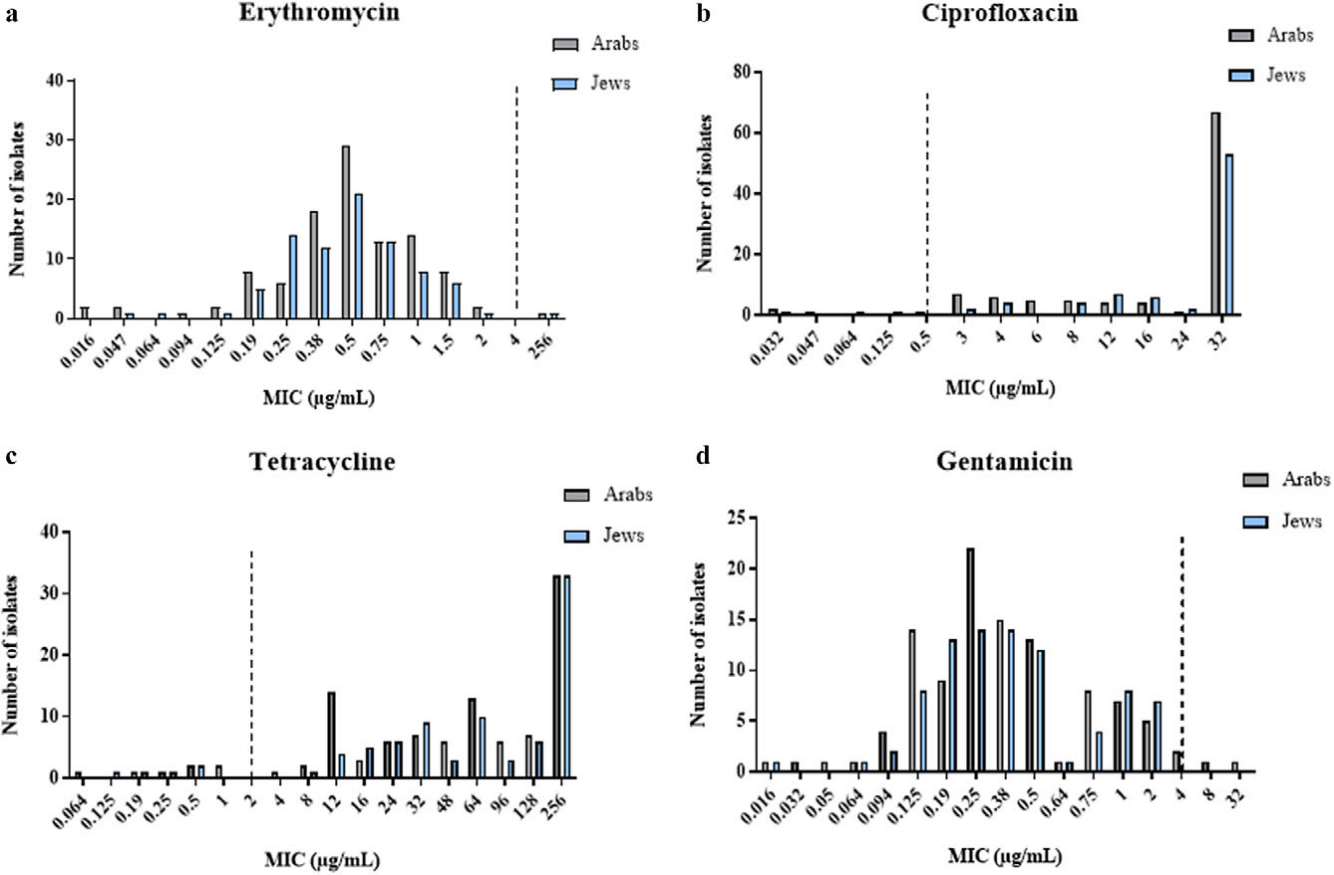
Distribution of minimum inhibitory concentrations (MICs) of antibiotics in *C.jejuni* isolates, by patient ethnicity. (a) Erythromycin, (b) ciprofloxacin, (c) tetracycline, and (d) gentamicin. The figure presents the antibiotic susceptibility of 191 *C. jejuni* isolates based on the E-test method.

Figures 2 and 3 show the distribution of MIC values of *C.ampylobacter coli* and *C.jejuni* isolates, respectively, categorized by patient ethnicity.

## Discussion

*C.coli* is one of the most common bacterial causes of gastroenteritis worldwide. According to an analysis conducted by the World Health Organization (WHO) Foodborne Disease Burden Epidemiology Reference Group (FERG), it was ranked the second-leading cause of foodborne infections, contributing to approximately 96 million cases worldwide [13]. In Israel, it is a highly significant causative agent of foodborne illnesses, reaching an incidence rate of 91/100,000 population in general and of approximately 363/100,000 in children under 1 year of age [14].

According to our study, the Arab patients were younger than the Jewish patients. Furthermore, a significant proportion (65.3%) of the Arab patients were under 2 years of age, while only 25% of the Jewish patients belonged to this age group (*p* < 0.001). Additionally, 41.3% of the Arab patients lived in villages compared to only 24 (24%) of the Jewish patients. According to a previous study carried out between 2012 and 2014 in our medical centre, Arab children, especially those residing in rural areas, had a greater incidence of campylobacteriosis than Jewish children [15]. Moreover, Jewish children who were infected with *C.coli* tended to be older than Arab children, which matches our results. The consistent pattern of ethnic predilection for this infection highlights the need for effective strategies to prevent and minimize its high morbidity among the Arab population in Israel.

It should be noted that Arab villages suffer from a considerable level of overcrowding, with many households accommodating multiple family members within a single living unit. This may contribute to the rapid transfer of *C.coli* strains in these regions. Moreover, in rural areas, particularly those inhabited by Arabs, the level of interaction with animals, particularly chicken, is quite high.

While *C.coli* infection can affect both children and adults, children under 5 years of age are more susceptible to the infection and may experience more severe symptoms than adults [16]. In developing countries, *C.coli* enteritis is almost exclusively considered a paediatric disease [17]. In the present analysis, 47.1% of *C.coli* infections were observed in children under 2 years of age, and 14.5% were found in children aged between 2 and 5 years.

A study conducted in Northeast Scotland found a higher incidence of *C.coli* infection in young children living in rural areas than in those living in urban areas, which is compatible with our findings [18]. Despite the fact that poultry are considered the main reservoirs of *C.coli* [19], the study associated the infection with direct exposure to farm animals and contaminated water, rather than to the consumption of poultry meat [19]. Another study that sampled chicken and turkey from various market chains in Israel found that 48% of the samples were contaminated with *C.coli* [20]. Moreover, a case–control study conducted on children in Spain showed that consuming chicken at least three times a week prior to the onset of symptoms increased the odds of acquiring campylobacteriosis six-fold [21]. Chicken is a popular food of choice in Israel and is widely available and consumed in various forms. Given the association between frequent consumption of chicken meals and the risk of campylobacteriosis, it is essential to implement preventive measures aimed at promoting the proper handling of chicken and its products, hand washing, and consumption of fruits and vegetables among children to control the spread of *C.coli* infections.

While most cases of campylobacteriosis self-resolve and can be managed with fluid replacement, antimicrobial therapy may be necessary in certain cases, particularly for patients with severe, prolonged, or systemic infections and for high-risk groups.

The emergence of resistant isolates highlights the significance of antimicrobial susceptibility testing and the crucial need for epidemiological surveillance. The epidemiology of antibiotic resistance in Israel is a complex issue that requires a multifaceted approach involving infection control, surveillance, and antibiotic stewardship. Our research found erythromycin resistance in only 0.5% of the study population, while resistance to ciprofloxacin, tetracycline, and gentamicin was observed in 95%, 93%, and 2.3% of the population, respectively. We did not identify any significant differences in antibiotic resistance neither between Arab and Jewish populations nor between adults and children. The resistance rate to erythromycin in the current study was markedly lower than that in other countries such as Ghana (96%) and Singapore (51%) [22, 23]. Furthermore, a review that analysed *C.coli* isolates from South America found an increase in the spread of erythromycin-resistant strains, raising concerns regarding the use of this antibiotic in both animals and humans [24]. The isolates showed widespread resistance to both ciprofloxacin and tetracycline, which matches our results [24]. Another surveillance study conducted in Italy between 2013 and 2016 also measured high resistance rates to both ciprofloxacin and tetracycline; 76% of the *C. jejuni* and 70% of the *C. coli* strains were ciprofloxacin-resistant, and 64% of all strains were tetracycline-resistant [25].

The highly elevated levels of resistance to tetracycline and ciprofloxacin pose significant challenges both locally and globally. In 2019, the Centers for Disease Control and Prevention (CDC) reported 448,400 infections and 70 deaths from drug-resistant *C.coli.* According to the report, there was a nearly two-fold increase in the proportion of *C.coli* strains exhibiting increased resistance to ciprofloxacin over the past two decades, which has restricted the treatment choices for patients [26]. In order to address the growing concern of antibiotic-resistant *C.coli*, it is important to implement good antibiotic stewardship practices and adopt alternative methods for controlling bacterial infections in both humans and animals.

One limitation of this study is that no investigation of the origin of the isolates was performed. A future study should assess the relatedness of these isolates to provide more comprehensive data on *C.coli* epidemiology. Another limitation is the missing data regarding poultry consumption and exposure to animals. However, this was a retrospective analysis, and therefore, we could not achieve these data.

In conclusion, the present analysis provided a comprehensive overview of the epidemiological and clinical characteristics and patient demographics of *C. jejuni* and *C. coli* infections in Northern Israel. The findings underscored the importance of implementing hygiene practices and sanitation protocols, adhering to food safety regulations and employing effective livestock management practices, undertaking key measures to ensure the safety of the food supply chain, and reducing the risk of infections among consumers. Furthermore, the study highlights the need for routine antibiotic resistance surveillance.

## Data Availability

The data that support the findings of this study are available from the corresponding author.
